# Identification of a Phytase Gene in Barley (*Hordeum vulgare* L.)

**DOI:** 10.1371/journal.pone.0018829

**Published:** 2011-04-21

**Authors:** Fei Dai, Long Qiu, Lingzhen Ye, Dezhi Wu, Meixue Zhou, Guoping Zhang

**Affiliations:** 1 Department of Agronomy, Zhejiang University, Hangzhou, China; 2 Tasmanian Institute of Agricultural Research, University of Tasmania, Launceston, Tasmania, Australia; Auburn University, United States of America

## Abstract

**Background:**

Endogenous phytase plays a crucial role in phytate degradation and is thus closely related to nutrient efficiency in barley products. The understanding of genetic information of phytase in barley can provide a useful tool for breeding new barley varieties with high phytase activity.

**Methodology/Principal Findings:**

Quantitative trait loci (QTL) analysis for phytase activity was conducted using a doubled haploid population. Phytase protein was purified and identified by the LC-ESI MS/MS Shotgun method. Purple acid phosphatase (PAP) gene was sequenced and the position was compared with the QTL controlling phytase activity. A major QTL for phytase activity was mapped to chromosome 5 H in barley. The gene controlling phytase activity in the region was named as *mqPhy.* The gene *HvPAP a* was mapped to the same position as *mqPhy*, supporting the colinearity between *HvPAP a* and *mqPhy*.

**Conclusions/Significance:**

It is the first report on QTLs for phytase activity and the results showed that *HvPAP a*, which shares a same position with the QTL, is a major phytase gene in barley grains.

## Introduction

Phytic acid, *myo*-inositol 1, 2, 3, 4, 5, 6-hexakisphosphate (InsP6), is a principle storage form of phosphorus (P) and inositol in cereal grains, and it is an effective polyanionic chelating agent [1]. Phytate deposition plays an important role in storage and homeostasis of both P and some other mineral nutrients during grain development and maturation [2]. However, phytic acid has been termed as an “anti-nutrient’’ because of its direct or indirect ability in binding minerals. Thus, phytic acid altered the solubility, functionality, digestibility and absorption of mineral nutrients, which significantly restrict the bio-availability of mineral nutrients in feed [3], [4]. Reduction of phytate levels or increase in phytase activity in plant seeds is an alternative strategy for improving nutrient efficiency in animal production [5].

Phytase (*myo*-inositol hexaphosphate hydrolase) hydrolyses phytic acid to *myo*-inositol and inorganic phosphate. Sandberg and Anderson [6] found that endogenous phytase in wheat bran played a crucial role in phytate degradation in the stomach and small intestine of humans. Moreover, phytate was degraded during food processing by enhancing natural phytase activity or by phytase pretreatment of legume and cereal grains [7]. Hence, the enhancement of endogenous phytase activity of cereal grains could improve the bioavailability of mineral nutrients in cereals [8], [9], [10]. Phytases are also considered as environment-friendly enzymes, by avoiding the additional supply of exogenous phosphate and reducing the phosphate pollution from agricultural animal waste [11], [12].

Phytases are widespread in nature, and relatively higher phytase activities have been reported in cereals, such as rye, wheat and barley [5], [13]. There are three groups of phytases based on the catalytic mechanism, i.e. histidine acid phosphatases or acid phosphatases, β-propeller phytases, and purple acid phosphatases (PAPs) [11], [14]. According to the position of their initial hydrolysis of phytate, phytases can also be classified as 3-phytases, 6-phytases or 5-phytases [11], [15]. Two main types of phytase have been identified in plants, acid phytase and alkaline phytase, with a pH optimum around pH 5 and 8, respectively [5]. Most of the purified phytases belong to the acidic ones, such as those from oat [16], maize [17] and faba beans [18]. Two types of phytases have been identified from 4-day-old barley seedlings. One phytase (P2) was identified as a constitutive enzyme, whereas the other one (P1) was induced during germination [13]. Several cDNAs encoding a group of enzymes with phytase activity in barley and wheat were cloned and characterized. They were named as multiple inositol phosphate phosphatases (MINPPs), all were acid phytase [19].

Purple acid phosphatases (PAPs) are widespread in mammals, fungi, bacteria, and plants, which are generally considered to mediate phosphorus acquisition and redistribution based on their ability to hydrolyze phosphorus compounds [20], [21]. All members of PAPs contain a characteristic set of seven amino-acid residues involved in metal ligation and a binuclear metallic center composed of two irons in animals, whereas one iron ion is replaced by either a zinc or manganese ion in plants [22]. Hegeman and Grabau [23] isolated a phytase gene from germinating soybean, which showed a high degree of sequence similarity to PAPs, named as *GmPHY*. The enzyme displayed optimal pH at 4.5–5.0. However, not all PAPs exhibit phytase activity and not all of these enzymes effectively utilized phytate as a substrate. In these cases, PAPs play an auxiliary role in the degradation of phytate [22].

Barley is an important food crop in many countries, as well as a basic material in both brewing and the feed industries [24], [25]. Many efforts have been made to improve phytase activity or reduce phytate concentration in edible tissues [2], [3], [6], [23]. Our previous studies showed that the phytic acid content in barley is greatly affected by both genetic and environmental factors [26], and phytase activity differed greatly among genotypes [27]. Therefore it is possible for us to reduce phytic acid content in barley products through improving phytase activity in grains.

This study aimed at identifying QTLs controlling phytase activity using a doubled haploid population; purifying and characterizing phytase proteins; and clarifying the relationship between the gene in the QTL region and different isoforms of purple acid phosphatase (PAP) gene.

## Materials and Methods

### Plant materials

A barley population consisted of 177 doubled haploid (DH) lines from a cross between Yerong and Franklin [28]. Franklin is an Australian two-rowed malting barley, and Yerong is an Australian six-rowed feed variety.

### Plant growth and sample preparation

The DH lines and parents were grown in four different environments. The first field trial was conducted at the farm of Zhejiang University, Huajiachi campus (ZUH), Hangzhou, China, in 2008–2009 growing seasons, with local-field management. The second and third field trials were conducted at ZUH in 2009–2010 growing seasons with two levels of nitrogen application. According to our previous report [29], 180 kg ha^−1^ and 120 kg ha^−1^ N was applied for High-Nitrogen (HN) and Low-Nitrogen (LN) treatments, respectively. Fifty percent of N as base fertilizer was applied before sowing, and twenty-five percent of N as urea was applied at booting and heading stage, respectively. All genotypes were sown in early November with adjacent plots in the field and each genotype consisted of 2-m-length row with 0.25 m between rows. Other field management was the same as applied locally. The fourth field trial was conducted at Forthside Vegetable Research Station (FVRS), Tasmania, Australia in 2007–2008 growing season. Each line was grown in a 2-m row plot with 0.4 m between rows. All agronomic management methods, including fertilization, weed and disease control, were in accordance with local practice.

Harvested grains were stored in a cool room at 4°C and were mixed and milled to pass through a 0.5 mm screen before analysis.

### Phytase activity assay

Phytase activity was analyzed according to the method reported previously [27], [30]. Phytic acid sodium salt hydrate (Sigma P0109 from rice) was used as substrate. For quick measurement, the liberated phosphorus was determined spectrophotometrically (700 nm) with Varioskan Flash Multimode Reader (Thermo Fisher Scientific, Waltham, MA, USA) in 96-well plates. The phytase activity was expressed as the amount of liberated inorganic phosphorus from sodium phytate solution at pH 5.5 and 37°C (1 unit = 1 µmol·min^−1^).

### QTL analysis

A genetic linkage map of Franklin/Yerong DH population was comprised of 496 DArT and 28 microsatellite markers [28]. QTLs were analyzed using the software package MapQTL5.0 [31]. QTLs were first analysed by interval mapping (IM). The closest marker at each putative QTL identified using interval mapping was selected as a cofactor and the selected markers were used as genetic background controls in the approximate multiple QTL model (MQM) of MapQTL5.0. Logarithm of the odds (LOD) threshold values applied to declare the presence of a QTL were estimated by performing the genome wide permutation tests with at least 1000 permutations of the original data set for the trait. Two LOD support intervals around each QTL were established, by taking the two positions, left and right of the peak, that had LOD values of two less than the maximum, after performing restricted MQM mapping which does not use markers close to the QTL. The percentage of variance explained by each QTL (R^2^) was obtained using restricted MQM mapping implemented with MapQTL5.0.

### Protein purification

Protein was extracted and purified according to previous reports [32], [33] with some modification. Five-hundred grams of barley flour (Franklin) was extracted with 2-L sodium acetate buffer (200 mM, with 5 mM Dithiothreitol, pH 5.0) for 1 h (stirred vigorously). The homogenate was centrifuged at 10000 g for 15 min, and the supernatant was collected for further concentration and enzyme activity measurements. The crude extract was used for ammonium sulfate precipitation at 50%–80% saturation and centrifuged at 10000 g for 15 min. The pellet was resuspended in 50 mM sodium acetate buffer (pH 5.0, with 20 mM CaCl_2_) and then dialyzed against the same buffer without CaCl_2_ overnight. Insoluble material was removed by centrifugation.

The enzyme was further purified by chromatographic procedures. All chromatography was carried out on a compact liquid chromatography system (ÄKTA primary plus, GE Healthcare, Sweden), sequentially with a cation-exchange column (HiTrap CM FF, 5 ml, GE Healthcare, Sweden), and a gel filtration column (HiPrep Sephacryl S-200 H, 16/60 mm, GE Healthcare, Sweden) following the manufacturers' instructions. The protein solution filtrated through 0.22 µm membrane was loaded onto a cation-exchange column at a flow rate of 5 mL min^−1^ that was equilibrated with 100 ml of pH 5.0 50 mM sodium acetate buffer (SAB). The column was rinsed with 100 mL of SAB and subsequently eluted by applying a linear gradient of 0 to 500 mM NaCl in the buffer (100 mL) at the same flow rate. The fractions containing phytase activity were pooled and concentrated using an Amicon Centrifugal Filter Unit (Millipore, Bedford, MA, USA). The concentrated protein solution filtrated through 0.22 µm membrane was then loaded onto a gel filtration column equilibrated with same buffer containing 150 mM NaCl. Protein was eluted at a flow rate of 0.5 mL min^−1^ and the fractions containing phytase activity were pooled. The purified protein was concentrated and stored at −80°C for further assays.

Eluted proteins were separated on one-dimensional 10% (w/v) SDS-PAGE and visualized by silver staining method [34]. A protein molecular weight standard was run in parallel to estimate the approximate molecular weights of the proteins separated.

### Protein identification

Proteins were identified using LC-ESI MS/MS Shotgun method [35]. All electrospray mass spectra were performed using a Finnigen LTQ VELOS mass spectrometer (Thermo Finnigan, San Jose, CA, USA). MS/MS raw data were used to search against the *Hordeum vulgare* protein database at NCBI (http://www.ncbi.nlm.nih.gov) using the SEQUEST algorithm incorporated into the BioWorks software (*Version 3.2*, Thermo Finnigan, San Jose, CA, USA).

### Protein determination

The protein content of the extracts was measured using a Bradford assay Kit (Bio-Rad Labs, Hercules, CA, USA) according to manufacturer's instructions, with bovine serum albumin as standard.

### DNA extraction, PCR amplification, and sequencing

Total genomic DNA was extracted from barley seedlings using Universal Genomic DNA Extraction Kit Ver.3.0 (TaKaRa Bio, Tokyo, Japan) as described previously [36]. Based on the cDNA sequences of Purple Acid Phosphatase retrieved from the NCBI database (*HvPAP a*: FJ974003 and *HvPAP b*: FJ974005, direct submitted by Dr. Dionisio in Aarhus University, Denmark), PCR primers were designed with the Primer 5.0 (File S1). Each 25 µl amplification reaction consisted of 2.5 µl 10×*TransTaq* HiFi Buffer I (200 mM Tris-HCl (pH 8.4), 200 mM KCl, 100 mM (NH_4_)_2_SO_4_, 20 mM MgCl_2_ ), 2 µl 2.5 mM dNTPs, 2 µl 10 µM primers, 0.5 µl 5 unit µl^−1^ of *TransTaq* polymerase High Fidelity (Beijing TransGen Biotech, Beijing, China), and 1 µl 50 ng of genomic DNA. All amplifications were performed on a DNA Engine Dyad thermal cycler (Bio-Rad Labs, Hercules, CA, USA). After the PCR product was purified, DNA sequencing was performed on an ABI 3730XL sequencer following the manufacturer's instructions (Applied Biosystems, Framingham, MA, USA). All results were conducted with two independent PCR products which have been deposited in the GenBank at NCBI (JF274704, JF274705).

### Statistical analysis

Each measurement was carried out with at least three replications. Statistical analysis was carried out by SPSS *v13.0* for windows (SPSS Inc, Chicago, Illinois, USA).

## Results

### Phytase property of Yerong and Franklin

**Figure 1:**
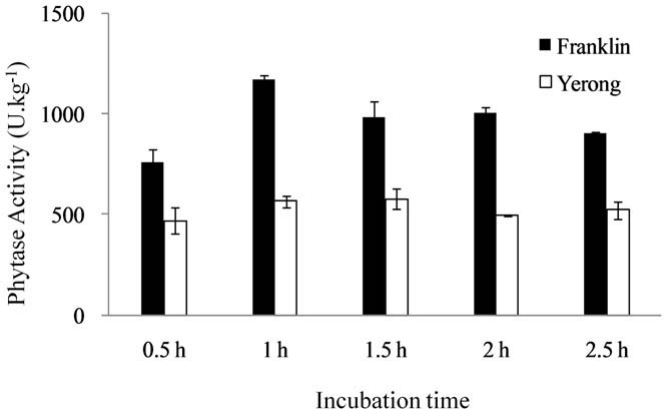
Effect of incubation time on phytase activity in Yerong and Franklin flours.

The phytase activity of Yerong and Franklin was determined after 0.5, 1.0, 1.5, 2.0 and 2.5 h incubation with substrate. Phytase in Franklin flour showed significantly higher activity after 1.0 h incubation. In contrast, incubation time had less effect on the phytase activity of Yerong flour (Fig. 1). The greatest difference in phytase activity between Franklin (1167.4 U·kg^−1^) and Yerong (565.2 U·kg^−1^) was found after 1 h incubation, thus this protocol was used in all further determinations.

To determine the similarity of phytase performance from Yerong and Franklin, mixed flour samples were prepared in different ratios (File S1). The predicted values of phytase activity were calculated according to the proportion of Yerong and Franklin. As shown in File S1, phytase activity of the mixed samples showed very close correlation with predicted values, suggesting the phytase in both Yerong and Franklin had similar performance in phytate degradation.

### Phenotypic variation among the DH lines of Franklin/Yerong

**Figure 2:**
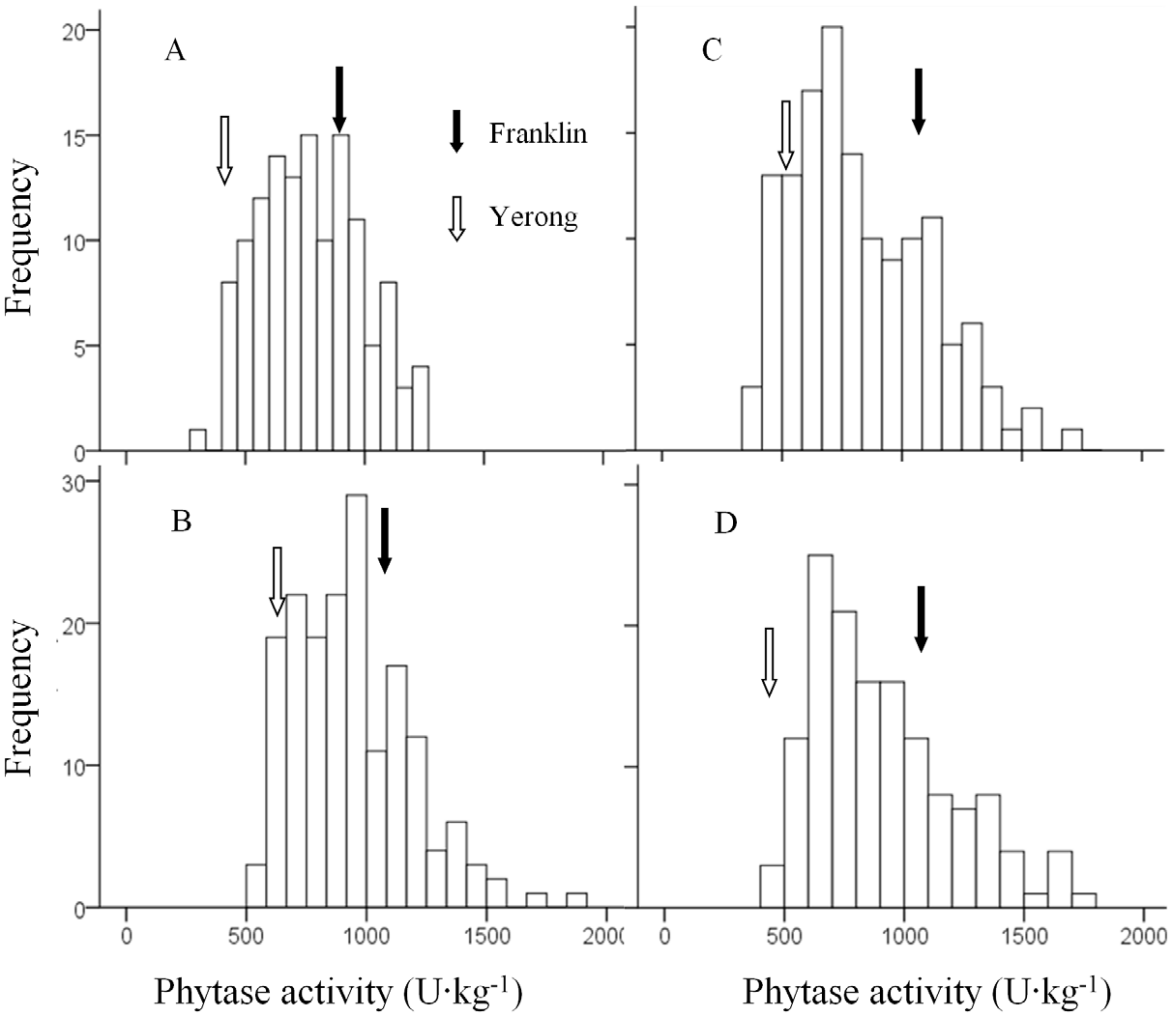
Frequency distribution for phytase activity in a DH population of Yerong/Franklin. A: the farm of Zhejiang University, Huajiachi campus (ZUH) in 2008–09 gorwing season; B: Forthside Vegetable Research Station (FVRS) in 2007–08 growing season; C and D: ZUH in 2009–10 growing season with High-Nitrogen (HN) and Low-Nitrogen (LN) application, respectively.

The distributions of phytase activity of the DH lines were shown in Fig. 2. Normal distributions were found for the samples from all different sites or treatments with no significant skewness and kurtosis. Transgression beyond the parental values was observed in all four sites or treatments. The coefficients of variation were similar among different sites or treatments (27.7, 25.9, 34.7 and 32.4 for ZUH, FVRS, ZUH-HN and ZUH-LN, respectively). The average phytase activity of DH lines were 774.1 U·kg^−1^, 940.3 U·kg^−1^, 825.9 U·kg^−1^ and 910.0 U·kg^−1^ for ZUH, FVRS, ZUH-HN and ZUH-LN, respectively. Samples from FVRS showed significantly higher phytase activity than those from other sites/treatments. High N treatment caused a significant reduction in phytase activity.

### Identification of QTLs associated with phytase activity

One QTL controlling phytase activity in barely grains was found on chromosome 5 H in both ZUH (*qPhy1.1*) and ZUH-HN (*qPhy3.1*) trials (Table 1) with the nearest marker being bPb-4334 and bPb-9476, respectively. The two-lod support intervals for the QTL detected in both trials were 58–67 cM. Two QTLs (*qPhy2.2, and qPhy4.2*) were identified in the other two trials. The major QTL was located at the same position as that identified in the ZUH and ZUH-HN trials, with the same 2-lod support intervals and bPb-9476 being the nearest marker. The minor QTL identified from both FVRS and ZUH-LN was located on chromosome 1 H with the 2-lod support intervals being around 44–76 cM. The major QTL identified from all the trials explained 30–47% of the phenotypic variation, indicating that the QTL in 5 H may be attributed to a gene controlling phytase activity in barely grains. This gene in the region was named as *mqPhy* (Table 1).

**Table 1:** QTLs for phytase activity in the DH population of Yerong/Franklin.

Site/Treatment	QTL	Chr.	Marker intervals	Nearest marker	Position (cM)	LOD	R^2^ (%)
ZUH	*qPhy1.1*	5 H	58–67	bPb-4334	65.4	9.78	29.5
FVRS	*qPhy2.1*	1 H	44.8–76	bPb-9334	56.6	2.95	4.8
	*qPhy2.2*	5 H	58–66	bPb-9476	58.9	17.45	35.0
ZUH-HN	*qPhy3.1*	5 H	58–66	bPb-9476	58.9	20.58	46.6
ZUH-LN	*qPhy4.1*	1 H	44–60	Bmag0090	51.6	5.2	8.7
	*qPhy4.2*	5 H	58–66	bPb-9476	58.9	19.41	41.9

Marker intervals are 2-lod support intervals around each QTL; the position is that of the nearest marker; R^2^ means percentage genetic variance explained by the nearest marker; FVRS: Forthside Vegetable Research Station; ZUH: farm of Zhejiang University, Huajiachi campus; HN and LN: High-Nitrogen and Low-Nitrogen application, respectively.

### Purification and identification of phytase

Phytase was purified from barley grains using three consecutive purification steps: selective precipitation with ammonium sulfate (50%–80%), a cation-exchange and a gel filtration column chromatography. The purification procedures of protein samples are illustrated in File S1. The phyase activity of the protein fraction at each step was determined with phytate as substrate. The fractions containing phytase activity were combined and used in the next steps. After the three-step purification, the phytase protein had been purified approximately 100-fold with an overall recovery of 10.1%, exhibiting a specific phytase activity of 3084.7 mU mg^−1^ protein (File S1).

The purified phytase solution (PPS) was fractionated by one-dimensional SDS-PAGE (10%). Two large polypeptide bands were detected by silver staining (File S1), approximately 60 and 45 kDa, respectively. The results suggested that there were several other proteins with similar properties as phytase in PPS. Thus LC-MS/MS spectrometry method was employed to indentify the proteins in PPS. The results showed that there were 11 groups of protein in the sample (Table 2). Interestingly, only one group of protein, identified by MASS spectrometry method (No. 3 in table 2), showed phytase activity according to NCBI protein database (http://www.ncbi.nlm.nih.gov), which was named as purple acid phosphatase (PAP).

**Table 2:** Proteins identified by the LC-MS/MS analysis in purified phytase solution.

No.	Protein name*	NCBI gi	Theoretical MW (kDa)	Theoretical PI
1	Lipoxygenase 1	2506825	96.4	5.7
2	Beta-amylase	144228332	59.6	5.6
3	Purple acid phosphatases	237847803	59.3	5.4
4	Unnamed protein product	296522893	54.6	8.5
5	Alanine aminotransferase 2	1703227	52.9	5.9
6	Elongation factor 1-alpha	6015054	49.1	9.2
7	Protein z-type serpin	1310677	43.2	5.6
8	Serpin-Z7	75282567	42.8	5.5
9	Fructose-bisphosphate aldolase	226316443	38.7	6.1
10	Beta-glucosidase	1683148	13.8	9.3
11	Chain B, Post Translational Modified Barley Ltp1	281307055	9.7	8.2

*Mass spectral data were searched against NCBI *Hordeum_vulgare* protein database.

### Sequencing and gene structure of *HvPAP a* and *HvPAP b*

**Figure 3:**
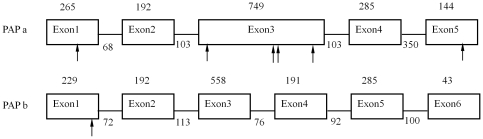
Gene structure and diversity of *HvPAP a* and *HvPAP b* in barley, with exons (boxes), introns (thin lines) and SNPs (arrows) found between Yerong and Franklin. Those two genes were based on the sequences of Franklin. For *HvPAP a* (Genebank: JF274704), the length of intron 2 and 3 was 105 and 109 bp for Yerong, respectively. The synonymous substitutions of *HvPAP a* were CAC/CAT, TAC/TAT, TCA/TCG, GAA/GAG, ACG/ACC and GTT/GTC from left to right for Franklin/Yerong, respectively. For *HvPAP b* (Genebank: JF274705), only part of exon1 and exon6 was sequenced for both cultivars with the synonymous substitution being CCT/CCC for Franklin/Yerong.

A NCBI database search revealed the presence of several cDNA for PAPs in barley. There are four isoforms of PAP genes in barley; *HvPAP a, HvPAP* b1 and b2, and *HvPAP c. HvPAP c* supposed to be expressed in chloroplasts. Thus, cDNA of *HvPAP a* and *HvPAP b* was selected for whole genome analysis (File S1). Since the primers used for sequencing both *HvPAP* b1 and b2 were the same, we will only use the term *HvPAP* b for both *HvPAP* b1 and b2 in this paper. The sequences of both *HvPAP a* and *HvPAP b* were obtained from NCBI database. Polymerase chain reaction (PCR) primers were designed based on these cDNA sequences (File S1). Sequence data of Yerong and Franklin were obtained from three (P1–1, P1–2 and P1–3) and two (P2–1 and P2–2) DNA fragments amplified using separate PCR reactions for *HvPAP a* and *HvPAP b*, respectively. Those two PAP genes in barley were highly homologous. Exon 2, 4 and 5 of *HvPAP a* and exon 2, 5 and 6 of *HvPAP b* shared the same length (Fig. 3). The exon 3 of *HvPAP a* shared the same length as the combination of exon 3 and 4 of *HvPAP b*, which was separated by intron 3 of *HvPAP b*.

The whole length of 2285 bp genomic DNA sequences of Franklin for *HvPAP a* was assembled (Genebank: JF274704), which consisted of five exons separated by four introns (Fig. 3). Six single nucleotide polymorphisms (SNPs) in the coding region were found between Yerong and Franklin (Fig. 3). All six base substitutions detected in the coding region were synonymous for the candidate genes in this study. More diversity was found in non-coding regions including SNPs and Indels between Yerong and Franklin (File S1).

A partial length of 1950 bp genomic DNA sequence for *HvPAP b* was assembled (Genebank: JF274705), without the first 21 and last 95 bp of cDNA. Six exons were separated by five introns for *HvPAP b* in the present results (Fig. 3). It seems that the *HvPAP b* gene is highly conserved in barley. Nucleotide sequence between Yerong and Franklin was identical, except for a synonymous SNP which was identified in exon 1 (Fig. 3).

### Physic mapping of *HvPAP a* and *HvPAP b*

**Figure 4:**
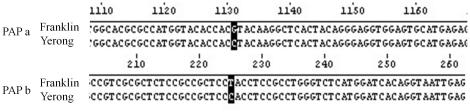
Single nucleotide polymorphism (SNP) of *HvPAP a* and *HvPAP b* used for SNP marker design between Yerong and Franklin.

**Figure 5:**
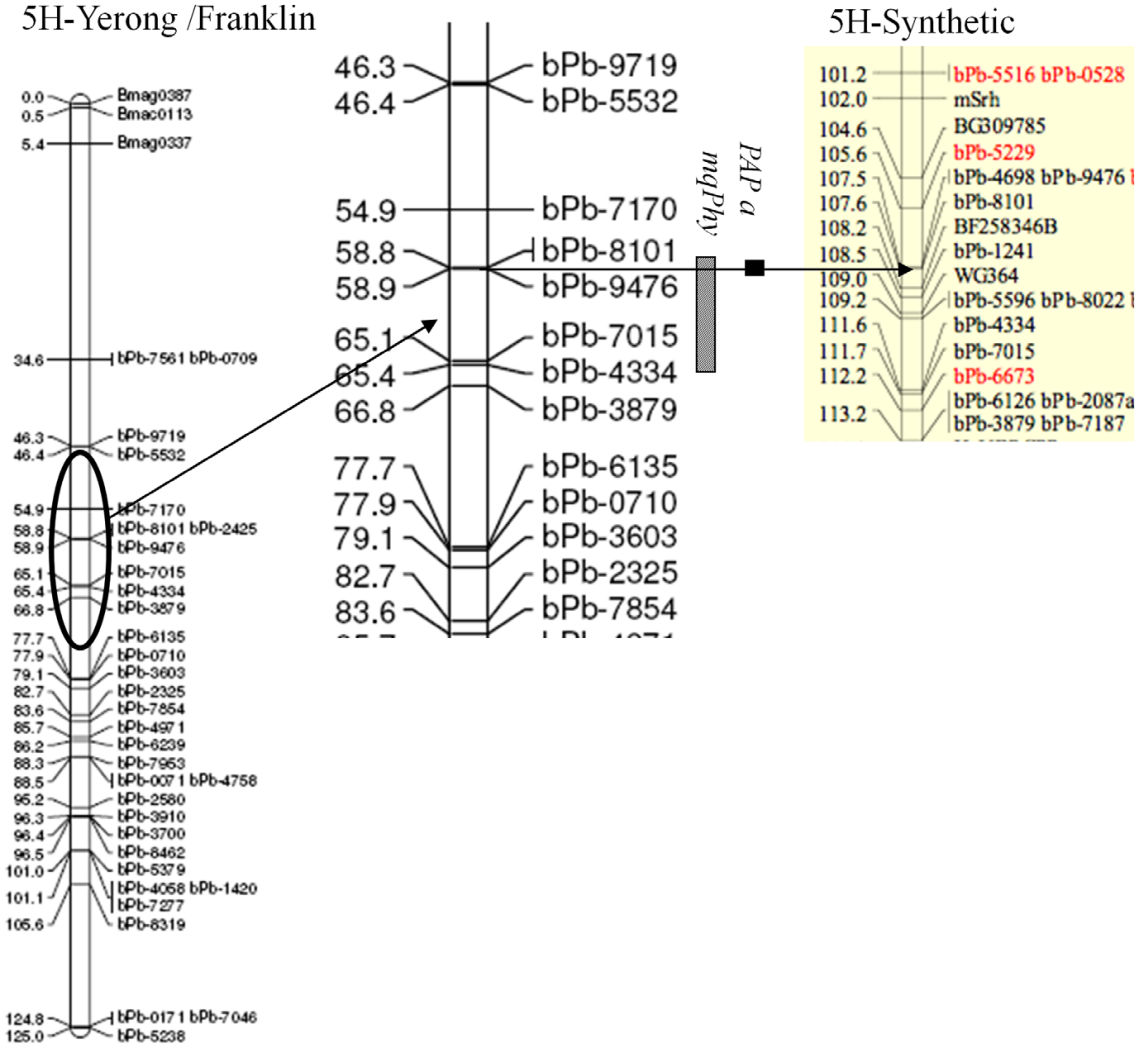
Quantitative trait loci (QTLs) identified for plant phytase activity in the DH population of Yerong/Franklin. This figure is for chromosome 5 H (Li et al., 2008). Part of synthetic map (Alsop et al., 2011) was added to the right for comparison. Arrows point out the position of closest DArT markers for *mqPhy* and *HvPAP a* genes in two different maps.

P1–4 primer, designed according to a SNP in exon3 of *HvPAP a* with a G/C substitution, was used to genotype 83 DH lines randomly selected from the Franklin/Yerong DH population, and P2–3 primer, designed according to **a** SNP in exon1 of *HvPAP b* with a T/C substitution, was used to genotype 117 DH lines from the same population (File S1, Fig. 4). No PCR product was obtained from Franklin for *HvPAP a*, and no *HvPAP b* from Yerong. *HvPAP b* was mapped to the chromosome 3 H at a position of 54.1 cM, proximal to marker Bmag0006. *HvPAP a* was mapped to the chromosome 5 H at position of 58.9 cM, proximal to marker bPb-9476 (Fig. 5), which was at the same position as the main QTL for phytase activity (*mqPhy*). The results of physical mapping provide the clear evidence to support the colinearity between the *HvPAP a* and *mqPhy*.

## Discussion

Phytases are widespread in nature, including microbes, plants and animal tissues. Various phytases have been isolated from plants, mainly grouped to acid phytase based on their pH optima, with an optimal pH at 4.5–6.0 [5], [37]. Some plant phytases are found to be purple acid phosphatases, and displayed optimal pH at 4.5–5.0 [23]. Phytases that catalyse the step-wise release of phosphate from phytate in plants usually show a very broad substrate specificity and a rather high affinity for phytate [5], [38]. It is suggested that phytases with broad substrate specificity are better suited for animal nutrition purposes than phytases with narrow substrate specificity [39]. A widely used method for estimation of phytase activity is to incubate the sample with phytate and estimate the phytase activity by determining released inorganic phosphorus, which can also be performed on crude extracted phytases [8], [27], [30], [40]. This method was employed in all the experiments in the current study.

Barley germplasm showed a wide genetic variation in phytase activity [27]. Based on our previous screening results, two barley genotypes, Yerong and Franklin, were used in the present study. Franklin showed much higher phytase activity than Yerong (Fig. 1). The results from samples with different ratios of Yerong and Franklin flours (File S1) showed that no other factors affected the phytase performance in barley grains when phytate was used as substrate. Different trial sites and/or N treatment also showed significant effects on phytase activity. High N treatment showed significant reduction in phytase activity, which may be related to the higher average protein content (86.8 mg·g^−1^) caused by the higher rate of N application compared to the average protein content of 77.4 mg·g^−1^ where a low rate of N was applied. Samples from FVRS showed the highest phytase activity, which may be due to the low protein content of the grains [41]. The low phosphorus content in Forthside soil which has very high P fixing capacity could also cause an elevated phytase activity as phytase is used to maintain adequate available P for growth of the plants with P deficient [42].

There have been no reports on QTLs controlling barley phytase activity (Gramene, http://www.gramene.org/qtl/). In the current study, a major QTL of phytase activity with high LOD score was identified in all four different sites/treatments. This QTL was located on chromosome 5 H with 2-LOD support intervals of 58–67. The gene controlling phytase activity was named as *mqPhy*. The position of this gene was at an equivalent position of 97.9 on the consensus map [43] (Fig. 5). A minor QTL on barley chromosome 1 H was also found in FVRS and ZUH-LN (Table 1), both sites showing low grain protein content. Since there is no significant correlation between phytase activity and protein content in barley grains [27], the small effect QTL in chromosome 1 H may be involved in low-level-N response. Further research is needed to clarify the effect of nitrogen application on phytase gene expression and enzyme activity. The small effect QTL could also be responsible to the transgressive segregation of progeny lines with higher or lower phytase activity than either parent being observed in different sites and/or N treatments. The additive effects from different QTLs could be beneficial in developing high phytase activity lines in barley.

Several phytases have been isolated from oat, spelt, maize, and barley [13], [16], [17]. One of them (P2) was identified as a constitutive enzyme, whereas the other one (P1) was induced during germination in barley [13]. The major difficulty encountered in phytase purification especially from plant sources is the separation of phytase from contaminating nonspecific acid phosphatases [44]. Since acid phosphatases are not capable of degrading phytate, the test of phytase activity is usually done with phytate as a substrate [13], which was used in the present study. Even though the purified phytase showed relative high activity (File S1), only a limited amount of phytase protein was obtained and it was still contaminated with several other proteins in PPS according to the SDS-PAGE examination (File S1). The MASS results showed that there were several groups of proteins (e.g. protein No. 2 named beta-amylase and No. 5 named alanine aminotransferase) with similar MW and *PI* to the phytase in PPS (Table 2). Only one group of proteins, purple acid phosphatase (PAP), which has phytase activity according to NCBI protein database (Table 2) was identified in this study, indicating that PAPs may play an important role as phytase in barley. The results are different from those previously reported by Dionisio, *et al.* [19], who suggested that the MINPPs should constitute an important part of the endogenous phytase potential in barley.

Purple acid phosphatases (PAPs) catalyze the hydrolysis of a wide range of activated phosphoric acid monoesters, diesters and anhydrides [45]. The adaptation of PAPs to degrade phytate may be a unique case in plants, although not all PAPs exhibit phytase activity [37]. Some PAP members can hydrolyze phytate to release inorganic phosphorus to be used in the germination of seed and pollen [33]. Several PAPs with phytase activity have been identified in soybean, tobacco and *Arabidopsis* [21], [23], [33].

A NCBI database search revealed the presence of several cDNA for PAPs in barley. Similar cDNA sequences are also available for wheat, *Arabidopsis*, rice and maize. However, there is no report on the structure and location of PAPs in barley. In the current study, cDNA of *HvPAP a* and *HvPAP b* was selected for whole genome analysis (File S1). Seven base substitutions were detected in the coding region which was synonymous for the two candidate genes, indicating that the difference of phytase activity between Yerong and Franklin may be attributed to the expression of those two genes instead of protein structure. Further studies are currently underway to determine the gene expression of PAPs in barley.

The comparison of DNA sequences from the two parent varieties revealed only one single nucleotide substitution of *HvPAP b* (Fig. 4). Similar results were found when comparing six barley genotypes, including three Tibetan annual wild barleys, with only one SNP in exon 1 and other two in non-coding region (data not shown). Mapping of this SNP was located in barley chromosome 3 H, which didn’t co-locate with any QTLs controlling phytase activity found in this study. *HvPAP a* was mapped to chromosome 5 H and at the same position where the *mqPhy* controlling phytase activity was located (Fig. 5).

In conclusion, a gene controlling phytase activity in barley was mapped to chromosome 5 H, and the phytase protein was purified from barley grains and identified as PAPs. Moreover, the gene *HvPAP a* was mapped to the same location. The current results will be helpful for barley breeders in developing new barley varieties with high phytase activity.

## Supporting Information

File S1 Supporting figures and tables.
(DOCX)
