# Supplementary material for: Identification of a Phytase Gene in Barley (Hordeum vulgare L.)
Source: PLoS One. 2011 Apr 21;6(4):e18829. doi: 10.1371/journal.pone.0018829 (PMC3080886; doi:10.1371/journal.pone.0018829)

Table S1 Primers used for sequencing and physical mapping of barley Purple Acid Phosphatase isoform a and b.

| Gene | Primer^2^ | Position^3^ | Forward sequence | Reverse sequence | Length (bp) |
| --- | --- | --- | --- | --- | --- |
| *HvPAP a* ^1^ | P1-1 | 17-893 | 5’-CAATGCCAAGCAACAACATC-3’ | 5’-TTGCCGATCTGCTCCTCTAT-3’ | 1032 |
|  | P1-2 | 771-1531 | 5’-GCCCATCCACGAAACCTACCA-3’ | 5’-GAGCGTGCGTCTCGTTCTTCA-3’ | 1214 |
|  | P1-3 | 860-1676 | 5’-AAGGGAACCACGAGATAGAGGAGC-3’ | 5’-CGAACCGTCTGTAGTTGCCATTTT-3’ | 1341 |
|  | P1-4 | 947-1151 | 5’-GGGTCCTTCTCCCCCTTCTA-3’ | 5’-TCCCTGTAGTGAGCCTTGTAG-3’ | 205 |
| *HvPAP b*^1^ | P2-1 | 25-799 | 5’-CTGCCGCTGTTTCTGCTT-3’ | 5’-CGGGCTCCATGTACCTTCC-3’ | 960 |
|  | P2-2 | 719-1522 | 5’-CCTGCTCGTTCGCCAAGT-3’ | 5’-GGTTCCTGTGCCATTTCCA-3’ | 1071 |
|  | P2-3 | 209-272 | 5’-CGCTCTCCGCCGCTCCT-3’ | 5’-CCGCCCATCTGGAAATCC-3’ | 136 |

1 The cDNA sequences were retrieved from the NCBI database (<http://www.ncbi.nlm.nih.gov>), *HvPAP a*: FJ974003 and *HvPAP b*: FJ974005.

2 PCR conditions: P1-1: 5 min at 94 ºC, 30 s at 94 ºC, 60 s at 58 ºC, 60 s at 72 ºC for 35 cycles, and 10 min at 72 ºC for a final extension; P1-2: 5 min at 94 ºC, followed by Touch-down PCR steps, 30 s at 94 ºC, 30 s at 60 ºC, decreased 0.2 ºC every cycle, 30 s at 72 ºC for 30 cycles, and 10 min at 72 ºC for a final extension; P1-3, P2-1, P2-2, P2-3: 5 min at 94 ºC, 30 s at 94 ºC, 90 s at 65 ºC (annealing and elongation) for 30 cycles, and 10 min at 72 ºC for a final extension; P1-4: 5 min at 94 ºC, 30 s at 94 ºC, 30 s at 65 ºC, 30 s at 72 ºC for 30 cycles, and 10 min at 72 ºC for a final extension.

3 This position was according to the cDNA sequence of *HvPAP a* and *HvPAP b*, respectively.

Table S2 Mixed sample of Yerong and Franklin and their phytase activity.

| Mixed Sample No. | 1 | 2 | 3 | 4 | 5 | 6 |
| --- | --- | --- | --- | --- | --- | --- |
| Franklin flour (%) | 100 | 80 | 60 | 40 | 20 | 0 |
| Yerong flour (%) | 0 | 20 | 40 | 60 | 80 | 100 |
| Detected value (U·kg^-1^) | 1167.4 | 1034.0 | 895.3 | 819.9 | 707.9 | 565.2 |
| Predicted value (U·kg^-1^) | 1167.4 | 1047.0 | 926.5 | 806.1 | 685.6 | 565.2 |

Table S3 Purification of Phytase from barley grains.

| Step | Volume  (ml) | Protein content  (mg ml^-1^) | Phytase Activity  (mU ml^-1^) | Specific Activity  (mU mg^-1^ protein) | Purification  (fold) |
| --- | --- | --- | --- | --- | --- |
| Crude extract | 1380 | 1.32 | 41.7 | 31.6 | 1 |
| AMS | 115 | 5.65 | 450.1 | 79.7 | 2.5 |
| HiTrap CM^*^ | 9.0 | 6.96 | 1666.7 | 239.5 | 7.6 |
| HiPrep S-200^*^ | 1.0 | 1.89 | 5830.0 | 3084.7 | 97.6 |

* The solution volume, protein content and phytase activity were measured after concentrating with Amicon Centrifugal Filter Units; AMS: Ammonium sulfate precipitation.

Figure S1. Correlation between detected and predicted values of phytase activity in mixed flour samples. The fraction of Yerong and Franklin was shown in Table S1; ^**^ significant at 0.01 probability level.

Figure S2. SDS-PAGE (10%) analysis of the purified protein solution (PPS) with silver staining. Lane 1: molecular mass standards; lane 2: sample. Approximately 2μg of protein was loaded.


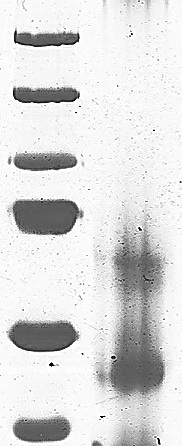


**2**

**1**

kDa

**250**

**150**

**100**

**75**

**50**

**37**

Figure S3. Single nucleotide polymorphism (SNPs) and Indels of *HvPAP a* in non-coding regions between Yerong and Franklin.

Franklin

Yerong

Intron 4


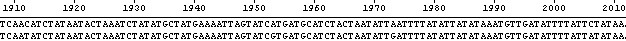

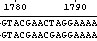

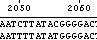

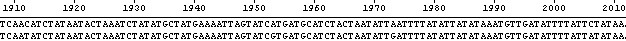


Franklin

Yerong

Intron 2


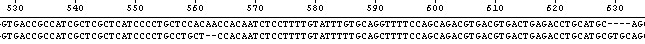


Franklin

Yerong

Intron 3


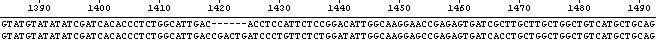

Supplement: File S1 — Supporting figures and tables. (DOCX) [file pone.0018829.s001.docx]
